# Multi-imaging study in a patient with cerebrotendinous xanthomatosis: radiology, clinic and pathology correlation of a rare condition

**DOI:** 10.1259/bjrcr.20190047

**Published:** 2020-02-12

**Authors:** Giuseppina Dell'Aversano Orabona, Clemente Dato, Mariano Oliva, Lorenzo Ugga, Maria Teresa Dotti, Mario Fratta, Pietro Gisonni

**Affiliations:** 1 Department of Advanced Biomedical Sciences, "Federico II" University of Naples, Naples, Italy; 2 Second Department of Neurology, "Luigi Vanvitelli" University of Campania, Naples, Italy; 3 Department of Neurology, IRCCS Policlinico San Donato, San Donato Milanese, Italy; 4 Department of Medicine, Surgery and Neuroscience, University of Siena, Siena, Italy

## Abstract

Cerebrotendinous xanthomatosis (CTX) is a rare metabolic disease with autosomal recessive inheritance. It is caused by mutations of the CYP27A1 gene, which codifies for sterol 27-hydroxylase, an enzyme that is responsible for the synthesis of cholic acids. In CTX, cholic acid synthesis is impaired, leading to accumulation of the precursor chenodessossicholic acid) in various organs and tissues. The clinical manifestations of CTX include chronic diarrhea, early-onset cataracts, tendon xanthomas and neurological disturbances. Therapy with oral chenodessossicholic acid has been shown to provide significantly better outcomes for affected individuals; therefore, recognition of this disease and awareness of its suggestive instrumental signs is extremely important. In this study, we describe the imaging findings in a 43-years-old male who was diagnosed with CTX and studied through ultrasound, CT and MRI.

It is important that the neurology and radiology communities are aware of this multi-imaging findings: recognition of them is important, as due to the high variability of the manifestation of this disease; it could impact on early diagnosis of a condition rarely seen, but manageable.

## Case report

### Clinical presentation

A 43-years-old male was brought to neurological evaluation for gait unsteadiness and frequent falls; these symptoms had had a subtle onset in the previous year and had progressively worsened since. His early medical history was notable for chronic diarrhea and developmental delay that had resulted in an intellectual disability. He was poorly educated and had never been employed; however, until the onset of these symptoms, he had been able to function in relative indipendence in his domestic environment. He was admitted for a full diagnostic assessment.

Physical examination showed macrocephaly and turricephaly, with multiple eyelid xanthelasmas. He presented with bilateral pedes cavi and a soft tissue mass protruding from his left Achilles tendon, suggestive of a tendon xanthoma. Neurological examination revealed an unsteady, paraparetic gait, that nonetheless allowed him to walk without support and up and down stairs. His lower limbs presented a slight degree of hyposthenia (Medical Research Council: 4/5) and spasticity (Ashworth scale: 1+/4), with bilateral tendon hyperreflexia. No overt signs of cerebellar involvement were detected, nor did he report any symptoms suggestive of such involvement, such as inability to perform fine hand movements.

Subtly progressive spastic paraparesis, consistent with pyramidal tract dysfuncton, may be a hallmark of neurodegenerative diseases such as hereditary spastic paraplegia and primary lateral sclerosis; other causes of chronic myelopathy, such as multiple sclerosis and spinal canal stenosis, were also ruled out.^1^

While the individual symptoms may be amenable to alternate explanations, their association was strongly suggestive of cerebrotendinous xanthomatosis (CTX).

## Imaging findings

### Brain MRI

Brain MRI showed initial signs of diffused cerebellar atrophy with volume loss (inappropriate to patient’s age) and ex-vacuo dilatation of fourth ventricle. Folia’s aspect was markedly prominent.

**Figure 1.**
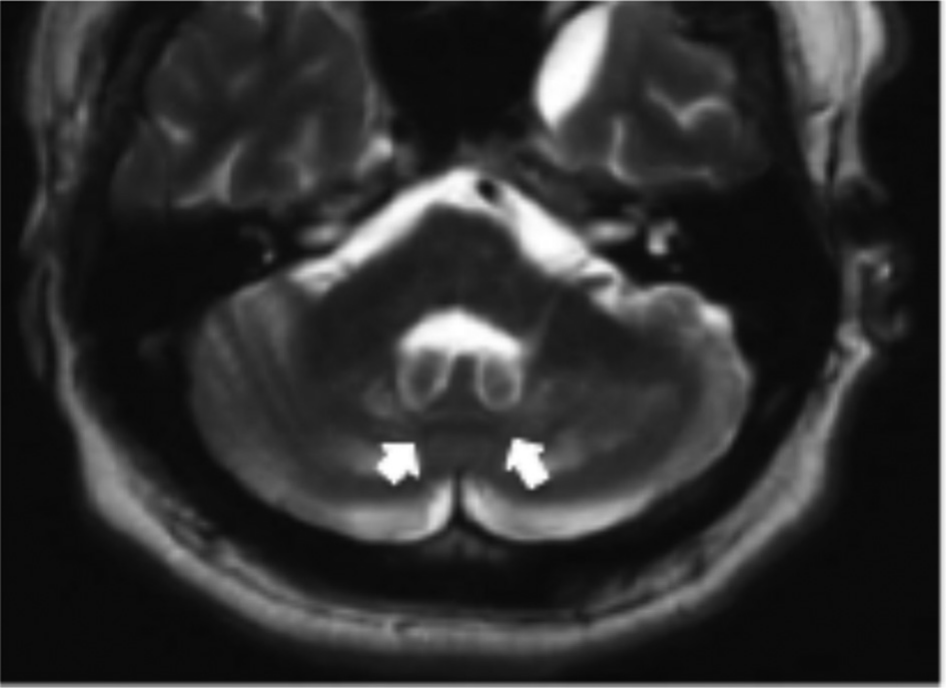
Axial *T*_2_W MRI of the brain shows bilateral hyperintense lesions involving the dentate nuclei (arrows) and the deep cerebellar white matter. The brain stem appears normal in signal intensity. *T*_2_W, *T*_2 _weighted.

*T*_2_ weighted (*T*_2_W) and fluid-attenuated inversion-recovery (FLAIR) images showed the symmetric hyperintensity of the dentate nuclei and of the cerebellar white matter with some associated calcium deposits in the adjacent zone (Figure 1). A soft hyperintensity on *T*_2_W and FLAIR was also noted in the superior cerebellar peduncles, that appeared thin (for gliotic phenomena), and, more feebly, in the pyramidal tracts.

Initial signs of cerebral atrophia were also presents with enlargement of the insular and frontal spaces.

### Neck CT findings and ColorDoppler ultrasound findings of carotid vessels

Angio-CT reported the presence of 15 mm atherosclerotic plaque, predominantly lipidic, at right carotid bulb which also involved the origin of internal carotid artery (ICA), producing a stenosis of 70%.

**Figure 2.**
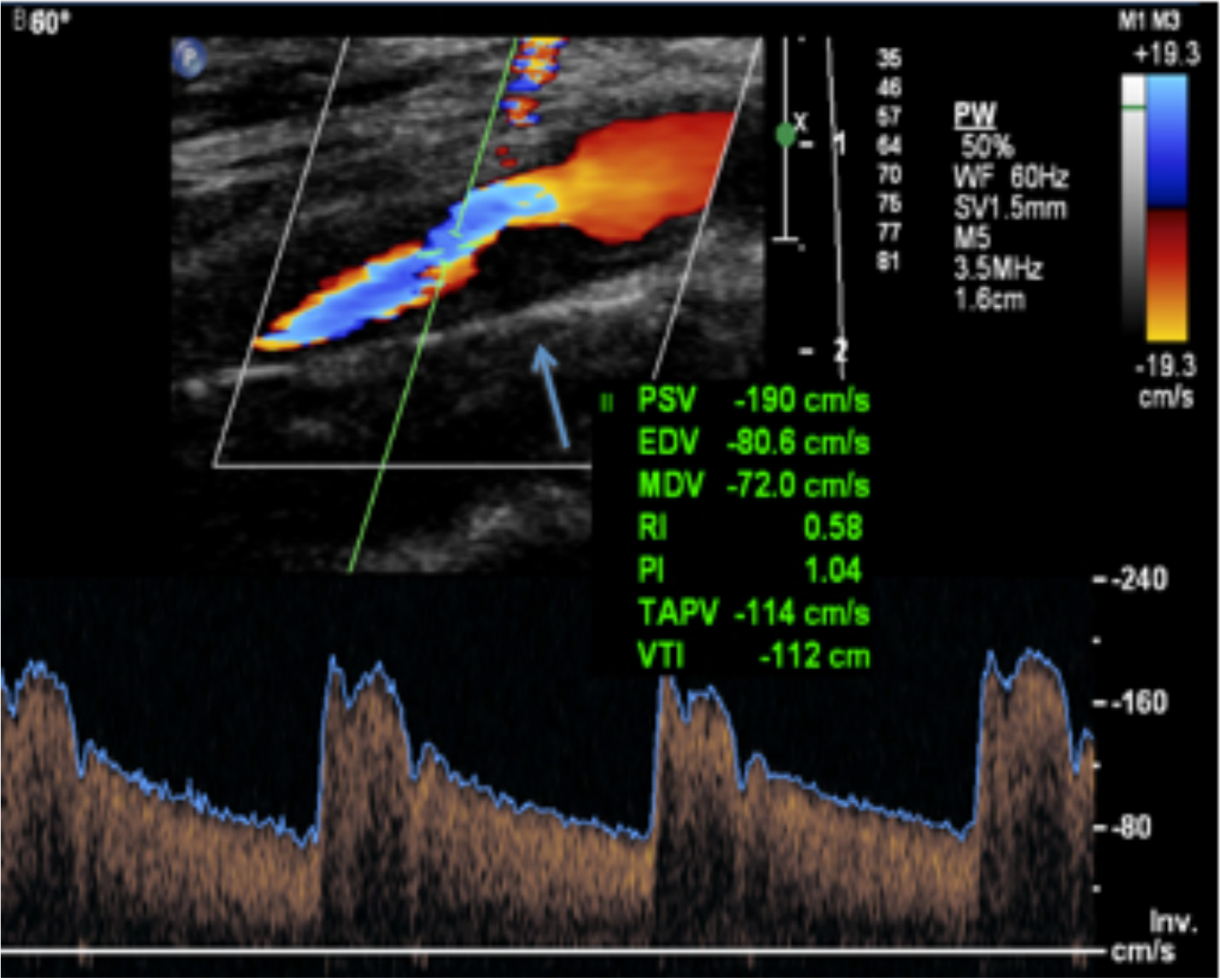
Color and Power Doppler ultrasound of right ICA with flow velocity analysis. According to NASCET criteria, a stenosis of 65–70% due to the presence of fibrocalcific plaque (arrow) was found, with SPV of 190 cm/s and DPV of 95 cm/s. DPV,diastolic peak velocity; ICA, internal carotid artery;SPV, systolic peak velocity.

Ultrasound examination of the neck vessels showed two fibrocalcific plaques of 2.2 mm in both carotid’s bulbes, which did not determine relevant alteration on the flow velocimetry. In correspondence of the right ICA was confirmed the stenosis of 65–70% according to NASCET with systolic peak velocity (SPV) of 190 cm/s and diastolic peak velocity (DPV) of 95 cm/s (Figure 2). No alterations of caliber and/or flussimetry in external carotid arteries and vertebral arteries.

### Achilles tendons ultrasound and MRI findings

**Figure 3.**
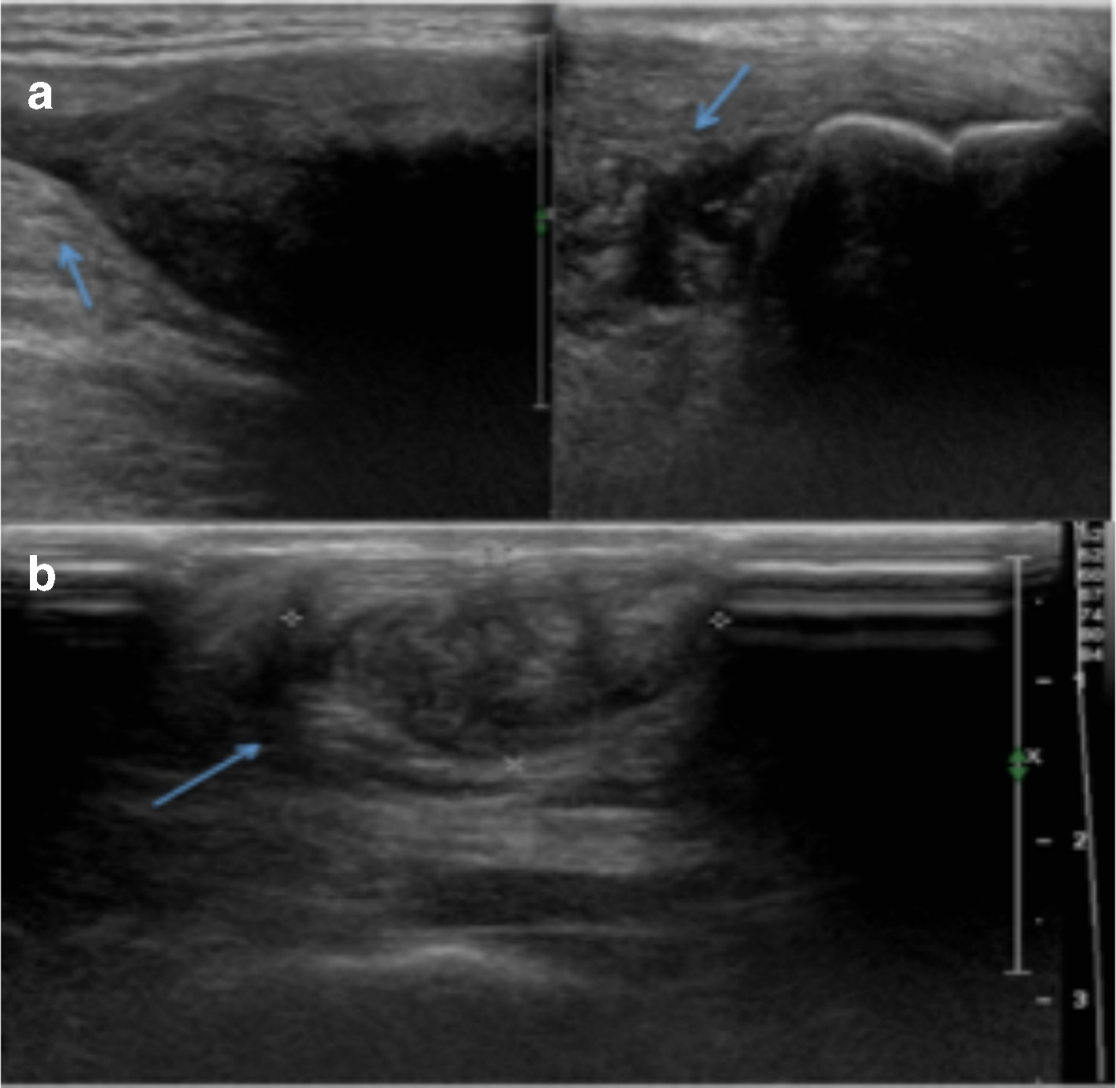
Ultrasound examination of Achille’s tendon. (a) Sagittal scan of both myotendinous and calcaneal side of tendon. Starting from anatomic extremities, fibrillar structures appeared opened wide with gradually enlargement of tendon’s body (arrow). (b) Axial scan shows structural upheaval of tendon (xanthoma) due to smooth hypoechoic intrafibrillar infiltration (arrow). In this scan is possible to obtain a reproducible measurement of xanthoma diameters.

**Figure 4.**
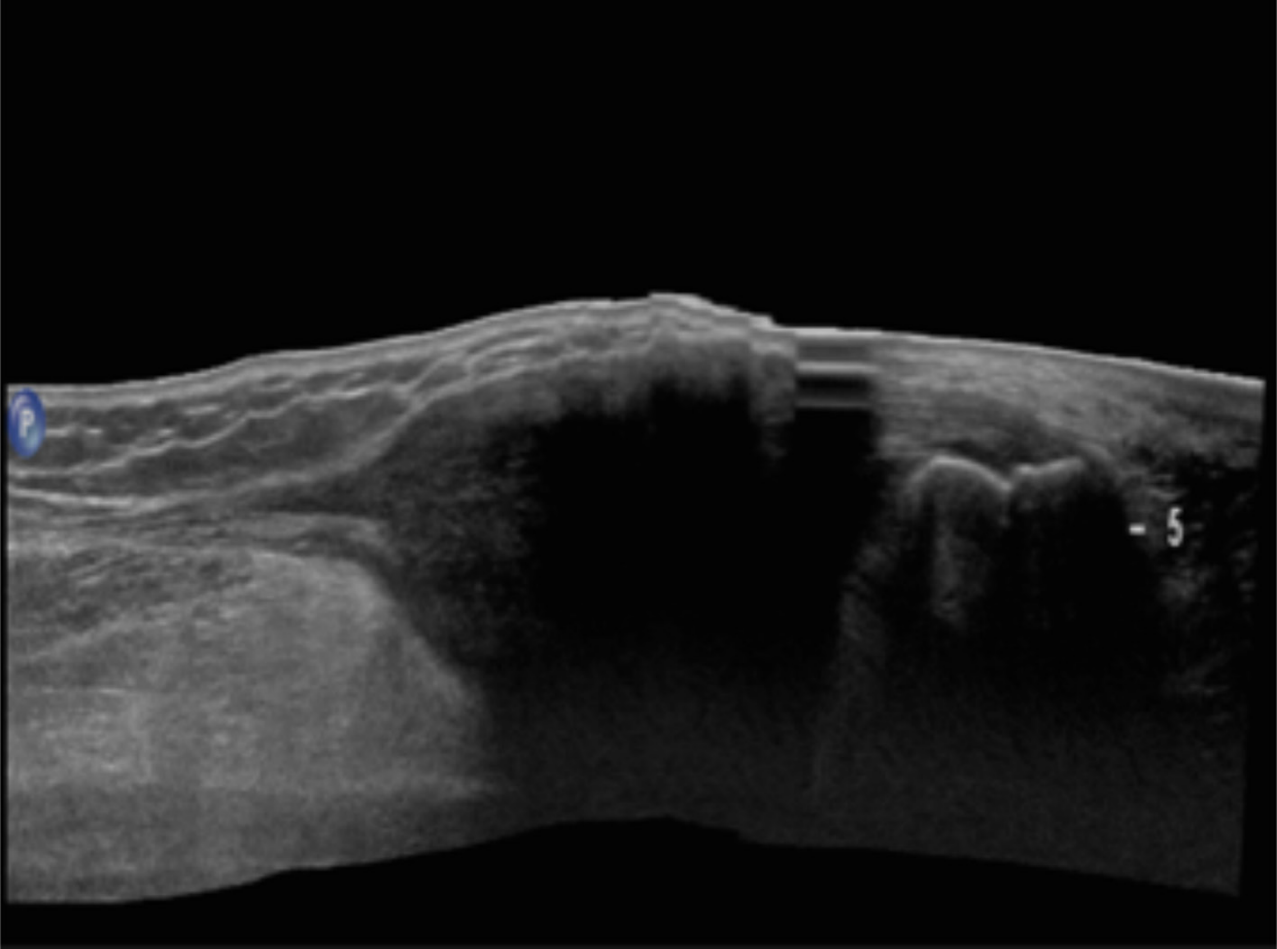
3D panoramic longitudinal scan of complete Achille’s tendon. In this image, it is possible to analyze tendon in length and note enlarged aspect. Tendon is quite completely visible and mostly thickened in the distal part. 3D, three-dimensional.

Ultrasound imaging of Achilles tendons (using a 5*–*12 MHz high frequency probe) was realized through axial and sagittal scans and showed enlarged tendons, mostly thickened in the distal part, with structural upheaval due to smooth hypoechoic intrafibrillar infiltration (Figures 3 and 4). The anteroposterior diameter of Achilles tendons was close to 21 mm on the right and 30 mm on the left. No alterations in correspondence of gastrocnemius and soleus insertions were found.

**Figure 5.**
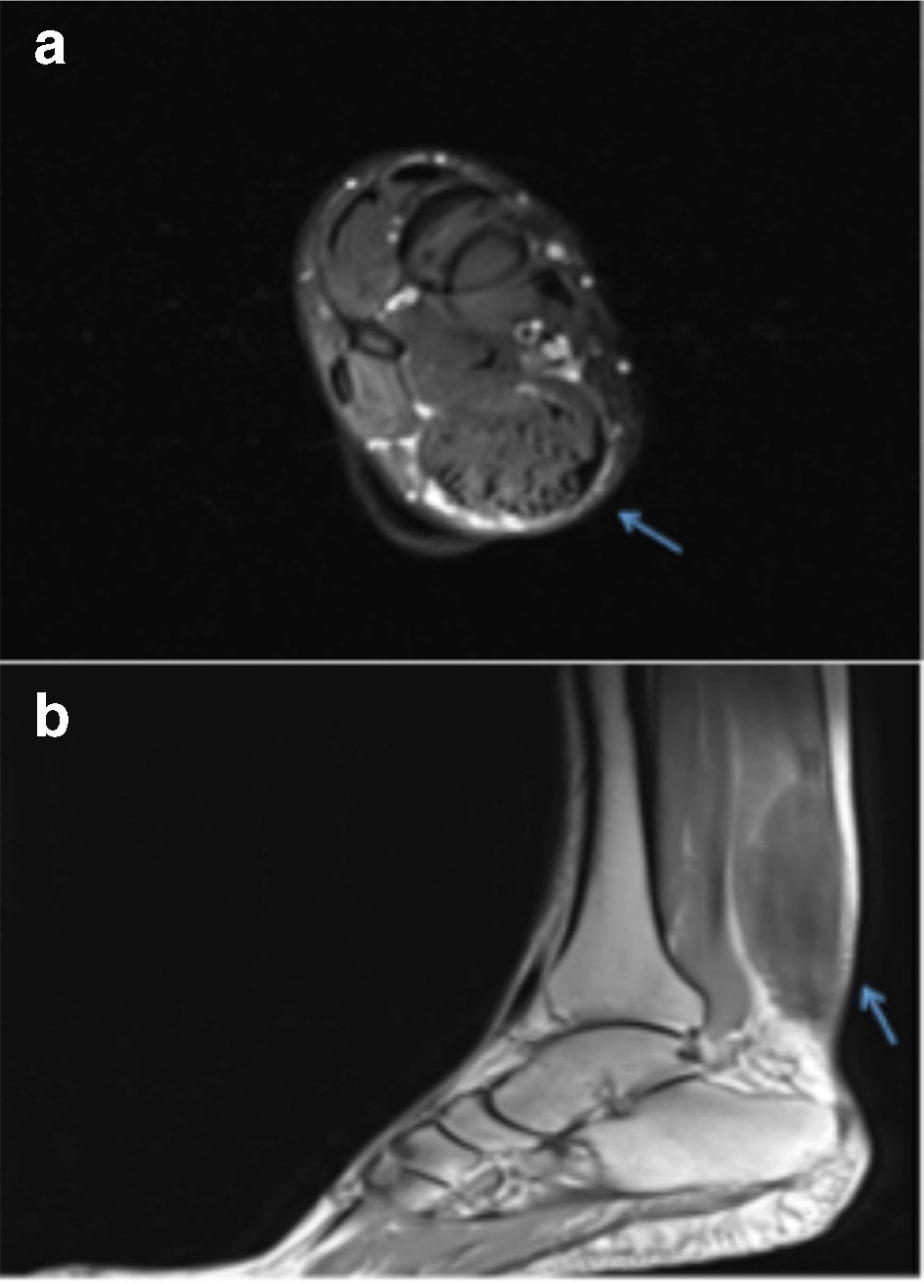
MR imaging of Achille’s tendon region. (a) *T*_2_ axial image. Tendon is visible in the posterior region of leg, appearing markedly enlarged and inhomogeneus for the presence of isointense areas alternated with rare smooth tendinous fibers (arrow). Note that lesion is well defined with smooth margins and no infiltration signs into the adjacent soft tissue are present. (b) *T*_1_ sagittal image. The sagittal projection effectively shows fusiform thickening of Achille’s tendon (arrow). It is possible to measure the craniocaudal length from the myotendinous junction to the calcaneal insertion and to study the relation with close structures and subcutaneous tissue.

MRI showed diffuse low intensity infiltration of Achilles tendons which appeared enlarged (thickness: 2,7 cm on the right side and 3,5 cm on the left side) and inhomogeneus for the presence of isointense (to the muscle) areas alternated with rare smooth tendinous fibers (Figure 5a). We measured the craniocaudal length (from the myotendinous junction to the calcaneal insertion) of this tendon’s fusiform thickening and it was equivalent to 12 cm on the right side and 10 cm on the left side (Figure 5b).

Both the lesions were well defined with smooth margins and no infilration signs into the adjacent soft tissue.

#### Genetic diagnosis

Given the findings, genetic analysis was performed, confirming the diagnosis of CTX (composed heterozygote for c.118*C* > A corresponding to p.Arg395Cys mutation and for a relatively recent mutation c.397*T* > C corresponding to p.Trp133Arg).

## Discussion

CTX is a rare metabolic disease with autosomal recessive inheritance.^1^ It is caused by mutations of the CYP27A1 gene, which codifies for sterol 27-hydroxylase, an enzyme that is responsible for the synthesis of cholic acids. In CTX, cholic acid synthesis is impaired, leading to accumulation of the precursor chenodessossicholic acid (CDCA) in various organs and tissues.^2^ The clinical manifestations of CTX include chronic diarrhea, early-onset cataracts, tendon xanthomas and neurological disturbances.^1^ Therapy with oral CDCA has been shown to provide significantly better outcomes for affected individuals.^3^

This rare lipid storage disorder, that was first identified by Van Bogaert in 1937,^4^ has an incidence estimated to be 0.13:100,000 individuals, as per 2018 Orphanet data^5^; however, novel genetic studies suggests it to be between 1 in 134,970 and 1 in 461,358 in Europeans and between 1 in 64,267 and 1 in 64,712 in East Asians.^6^ These recent advancements describe CTX as a rare, but also underdiagnosed condition. As more data about the effectiveness of early treatment on affected individuals become available,^3^ it underlines the importance of recognizing this disease through its clinical and instrumental signs.

CTX is caused by a mutation of the CYP27A1 gene, located on chromosome 2.^2^ CYP27A1 codifies for the sterol 27-hydroxylase, a hepatic mitochondrial enzyme that is responsible for the synthesis of biliary acids. In individuals with CTX, almost no CDCA is produced^1^; this leads to accumulation of cholesterol and its by-product, cholestanol, in tendons, in neurons, vascular subendothelial space and blood.

Biochemical diagnosis of CXT is based on elevated serum cholestanol levels and elevated urine bile alcohol levels.^2,7^

Mainstay of diagnosis is clinical suspicion, imaging and biochemical findings.

*Tendon xanthomas* are the most classical finding of CTX and may become appearent in the second or third decade. There may also be involvement of the extensor tendons of the elbow and hand, of the patellar tendon, and the neck tendons. Xanthoma formation has also been described in the lung, bones, and central nervous system.^1^ Furthermore, lipid desposition is also responsible for the appearance, of palpebral xanthelasma.^8^

In our case, ultrasound examination of the Achilles tendon was performed prior to the MRI study of the same district. It revealed loss of the normal fibrillary architecture of both tendons with thickening and smooth, symmetric, hypoechoic infiltration. The sonographic criteria for abnormal Achilles tendons have been described. Generally, tendons were considered enlarged if the maximum anteroposterior diameter exceeded two standard deviations above a mean of 5.7 + 7.1 mm, as determined by Steinmetz et al.^9^ In our case, the maximum anteroposterior diameter was 21 mm on the right side and 30 mm on the left side. Xanthomas appear as focal or confluent hypoechoic regions sonographically.^10^

Familial hypercholesterolemia is the most common state associated with tendinous xanthomatosis with tendon xanthomas developement in childhood (homozygous FH patients) or around 20 years old (heterozygous FH patients). Achille’s tendon xanthomatosis could be associated to some types of hyperlipidemia and chromosome mutations (drug-induced hyperlipidemia for antiretroviral therapy or familial recessive hypercholesterolemia) or to non-familial hypercholesterolaemia (as diabetes mellitus with hyperlipidemia and tissue accumulation of lipids). Although tendon xanthomatosis is very rare in normolipidemic patients, it should be known that in some cases there is not association with elevated low-density lipoprotein levels, *e.g.* in plexiform xanthomatous tumour, generally located on the knee, foot, hand or Achilles tendon.

In order to obtain the correct diagnosis, it is important to interpret Achilles tendon thickening as cholesterol accumulation only after exclusion of other disorders like tendonitis, peritendinitis, bursitis, trauma, nodules from rheumatic arthritis or gout tophi. Differential diagnosis with ATX may be based on detailed medical history, ultrasonography of the affected tendon and other biochemical examinations.^11^

Several studies demonstrated that high-resolution ultrasound is as sensitive as MRI in depicting the number and extent of intratendinous lesions. CT scan and ultrasound have been used to monitor the response to treatment as both are equally good in assessing the anteroposterior diameter of the tendons, the reduction of wich is a parameter for measuring the success of therapy.^12^ It is preferable to use ultrasound due to lack of radiation and equal efficiency as useful marker of treatment response.^13^

*Atherosclerosis* is prevalent and premature in patients with CTX, and may present as coronary, carotid or peripheral artery disease.^1^ It can be partially explained by alteration of the plasma lipid profile and lack of sterol 27-hydroxylase activity in the cellular components of the atheroma, which leads to cholestanol accumulation in the vascular subendothelial space.^2^ Several patients with CTX develop premature atherosclerosis and consequent cardiac events.^14^

In our case, angio-CT images of our patient showed premature atherosclerosis with evidence of a lipidic plaque at the origin of internal carotid artery. Ultrasound analysis of flow confirmed the percentage of stenosis. The finding of 70% stenosis in a 40-years-old individual with no other risk factor was a striking occurrence, and the one most likely to require immediate medical attention.

*Neurological presentation* of CTX is thought to be due to the accumulation of cholestanol in the brain and cerebrospinal fluid.^1^ Though it is not known how accumulation of cholestan produces functional abnormalities, the presence of apolipoprotein B in cerebrospinal fluid indicates penetration of low-density lipoprotein particles from plasma through the blood*–*brain barrier.^8^ Clinical expression is extremely variable in its characterics and age of onset.^1^ Some patients have a delayed cognitive and motor development, and may remain intellectually disabled. In most cases, however, neurological signs and symptoms start in the adult life (third to fourth decade), with behavioral disturbances and progressive cognitive decline, which may progress to dementia. Also typical of the adult age is a spectrum of movement disorders, ranging from spastic paraparesis, to ataxia, to atypical parkinsonism with dystonia. Epileptic seizures have been described in 50% of patients.^1^ Dysmyelinizing polyneuropathy may present with pes cavus and distal muscle atrophy.

In the case here described, CTX is most likely responsible for developmental delay, bilateral pes cavus, and of the late-onset spastic paraparesis.

Brain MRI in CTX reveals *T*_2_W hyperintensities in the dentate nucleus and in the deep cerebellar white matter, with associated calcium deposits that are due to the accumulation of sterols in the nerve cells, along with varying degrees of demyelination. The most frequent neuroimaging findings in patients with CTX are nonspecific cerebral and cerebellar atrophy and periventricular white matter lesions while more characteristic lesion is seen in the basal ganglia, cerebral peduncles, and dentate nuclei.^15,16^

Other findings includes thin appearance of superior cerebellar peduncles, softly hyperintensity of pyramidal tracts and signs of generalized atrophy of cerebellum hemispheres. Cerebellar involvement typically starts in the dentate nucleus and may eventually expand to the surrounding white matter of the cerebellar hemispheres.

Differential diagnoses for bilateral dentate nucleus *T*_2_ hyperintensity include metronidazole central nervous system toxicity in which, however, patients present with acute encephalopathy symptoms (cerebellar dysfunction, altered mental state and/or seizures). Langerhans cell histiocytosis represents another condition that can show similar MR findings. In these patients, the clinical history and the absence of tendon involvement can help to guide the diagnosis.^17^

In our patient, these lesions consisted of areas of high signal intensity on *T*_2_W MR images and were isointense or, when extensive, hypointense on *T*_1_W images, according to the literature.^15^

A combination of bilateral dentate nucleus hyperintensity with tendon xanthomas is almost diagnostic.

### Treatment

Treatment with CDCA lowers the concentration of cholestanol and other abnormal metabolites in body fluids; more importantly, it stabilizes the progression of neurological signs and symptoms.^3^

The basis of treatment with chenodeoxycholic acid is negative feedback for the bile acid biosynthesis pathway which suppresses production of cholestanol and bile alcohols.^18,19^

CDCA is administered orally, with a total dosage of 750 mg/day in adults; therapy must be continued life-long.^1^ The earlier treatment with CDCA is started, the more effective it is at preventing neurologic damage and deterioration. Patients who start treatment after 25 years may continue to clinically deteriorate despite improvement in biochemical parameters.^20^

Statins are able to control hypercholesterolemia, and are often prescribed for their pleiotropic effects.^1^

Other symptomatic treatments may be prescribed, according to the clinical presentation. Cataracts may require surgical extraction. Physical therapy and appropriate drugs may be prescribed to counteract gait imbalances, parkinsonism or spasticity.^1^

## Learning points

In this study, we elucidated the instrumental signs associated with a case of CTX that we studied through US, CT and MRI.

- Awareness and recognition of these imaging findings is important because they may be seen by professionals in any area of clinical expertise.
- A correct assessment is necessary in order to formulate and diagnose this rare condition, which in turn allows for early treatment and genetic screening of the relatives.
- Therefore, ultrasound can be used as sensitive tool for close monitoring of treatment response and post-therapy follow-up without radiation dose.
